# Oral Co-Supplementation of Curcumin, Quercetin, and Vitamin D3 as an Adjuvant Therapy for Mild to Moderate Symptoms of COVID-19—Results From a Pilot Open-Label, Randomized Controlled Trial

**DOI:** 10.3389/fphar.2022.898062

**Published:** 2022-06-07

**Authors:** Amjad Khan, Somia Iqtadar, Sami Ullah Mumtaz, Michael Heinrich, Domingo A. Pascual-Figal, Shona Livingstone, Sajid Abaidullah

**Affiliations:** ^1^INEOS Oxford Institute for AMR Research, University of Oxford, Oxford, United Kingdom; ^2^Department of Biochemistry, Liaquat University of Medical and Health Sciences, Jamshoro, Pakistan; ^3^Department of Medicine, King Edward Medical University, Lahore, Pakistan; ^4^UCL School of Pharmacy, University of London, London, United Kingdom; ^5^Department of Cardiology, University of Murcia Hospital Universitario Virgen de la Arrixaca Murcia, Murcia, Spain; ^6^Centro Nacional de Investigaciones Cardiovasculares (CNIC), Madrid, Spain; ^7^School of medicine, University of Dundee, Dundee, United Kingdom

**Keywords:** COVID-19, curcumin, quercetin, vitamin D3, anti-inflammatory, antiviral

## Abstract

**Background:** Curcumin, quercetin, and vitamin D3 (cholecalciferol) are common natural ingredients of human nutrition and reportedly exhibit promising anti-inflammatory, immunomodulatory, broad-spectrum antiviral, and antioxidant activities.

**Objective:** The present study aimed to investigate the possible therapeutic benefits of a single oral formulation containing supplements curcumin, quercetin, and cholecalciferol (combinedly referred to here as CQC) as an adjuvant therapy for early-stage of symptomatic coronavirus disease 2019 (COVID-19) in a pilot open-label, randomized controlled trial conducted at Mayo Hospital, King Edward Medical University, Lahore, Pakistan.

**Methods:** Reverse transcriptase polymerase chain reaction (RT-PCR) confirmed, mild to moderate symptomatic COVID-19 outpatients were randomized to receive either the standard of care (SOC) (*n* = 25) (control arm) or a daily oral co-supplementation of 168 mg curcumin, 260 mg quercetin, and 9 µg (360 IU) of cholecalciferol, as two oral soft capsules b.i.d. as an add-on to the SOC (*n* = 25) (CQC arm) for 14 days. The SOC includes paracetamol with or without antibiotic (azithromycin). Severe acute respiratory syndrome coronavirus 2 (SARS-CoV-2) RT-PCR test, acute symptoms, and biochemistry including C-reactive protein (CRP), D-dimer, lactate dehydrogenase, ferritin, and complete blood count were evaluated at baseline and follow-up day seven.

**Results:** Patients who received the CQC adjuvant therapy showed expedited negativization of the SARS-CoV-2 RT-PCR test, i.e., 15 (60.0%) vs. five (20.0%) of the control arm, *p* = 0.009. COVID-19- associated acute symptoms were rapidly resolved in the CQC arm, i.e., 15 (60.0%) vs. 10 (40.0%) of the control arm, *p* = 0.154. Patients in the CQC arm experienced a greater fall in serum CRP levels, i.e., from (median (IQR) 34.0 (21.0, 45.0) to 11.0 (5.0, 16.0) mg/dl as compared to the control arm, i.e., from 36.0 (28.0, 47.0) to 22.0 (15.0, 25.0) mg/dl, *p* = 0.006. The adjuvant therapy of co-supplementation of CQC was safe and well-tolerated by all 25 patients and no treatment-emergent effects, complications, side effects, or serious adverse events were reported.

**Conclusion:** The co-supplementation of CQC may possibly have a therapeutic role in the early stage of COVID-19 infection including speedy negativization of the SARS-CoV-2 RT-PCR test, resolution of acute symptoms, and modulation of the hyperinflammatory response. In combination with routine care, the adjuvant co-supplementation of CQC may possibly help in the speedy recovery from early-stage mild to moderate symptoms of COVID-19. Further research is warranted.

**Clinical Trial Registration:** Clinicaltrials.gov, identifier NCT05130671

## Introduction

Systemic hyper inflammation, prominent in the lungs and vascular endothelium, is the hallmark of severe coronavirus disease 2019 (COVID-19). There is currently no specific and conclusively proved treatment available for COVID-19. Anti-inflammatory (immunomodulatory) agents including corticosteroids (W.H.O. Rapid Evidence Appraisal for COVID-19 Therapies Working Group, 2020; RECOVERY Collaborative Group, 2021a) and interleukin (IL)-6 receptor inhibitor tocilizumab (RECOVERY Collaborative Group, 2021b) have been shown to reduce mortality, while Janus kinase (JAK) inhibitors (Kalil et al., 2021) aid rapid clinical improvement in severe COVID-19 illness. Recently, molnupiravir, sotrovimab, casirvimab/imdevimab, and nirmatrelvir/ritonavir have emerged as an early-stage COVID-19 antiviral therapies; however, these medications are costly and only available in the United Kingdom and the United States of America. A combination of early-stage therapeutic interventions that can interfere with the severe acute respiratory syndrome coronavirus 2 (SARS-CoV-2) replication and simultaneously modulate the host’s hyperinflammatory immune response, is considered “crucial” in preventing the progression to severe COVID-19 illness. There is currently an urgent need for safe, effective, and affordable therapeutic agents that are available worldwide to help manage the early-stage COVID-19 infection in vulnerable patients. In the present circumstances, integrative medications with anti-inflammatory, immunomodulatory, or antiviral scientific rationale may offer a suitable choice of treatment of early-stage COVID-19 infection. Among such complementary agents, natural polyphenols curcumin, quercetin, and vitamin D3 (cholecalciferol), hereafter combinedly referred to as CQC, have attracted scientific interest as possible early-stage adjuvant therapies for COVID-19 due to their pharmacological effects.

Curcumin, the yellow pigment of the spice turmeric, is the major active polyphenolic compound extracted from the roots of the rhizome plant *Curcuma longa L.* (Zingiberaceae). Curcumin has a wide range of *in vivo* pharmacological activities such as anti-inflammatory, antiviral, antioxidant, antimicrobial, antithrombotic, antitumor, cardioprotective, and hepatoprotective (Aggarwal and Sung, 2009; Bandyopadhyay, 2014; Nabavi et al., 2014; Perrone et al., 2015; Padmanaban and Rangarajan, 2016; Kunnumakkara et al., 2017; Boroumand et al., 2018; Giordano and Tommonaro, 2019; Karimi et al., 2019; Patel et al., 2020). For centuries, turmeric has been used in Ayurveda, Siddha, and Traditional Chinese Medicines to treat diverse diseases associated with infection and inflammation. Curcumin is also an effective antiviral agent against many viruses including dengue virus, human immunodeficiency virus (HIV), Kaposi sarcoma-associated herpesvirus, enterovirus, Zika virus, Chikungunya virus, vesicular stomatitis virus, human respiratory syncytial virus, influenza A virus, hemorrhagic septicemia virus, herpes simplex type 2, norovirus, hepatitis C virus, hepatitis B virus, SARS-CoV-1, and SARS-CoV-2 (Wen et al., 2007; Praditya et al., 2019; Jennings and Parks, 2020; Bormann et al., 2021; Marin-Palma et al., 2021; Bahun et al., 2022). Curcuminoids have an excellent safety profile with “Generally Recognized As Safe” (GRAS) status received from the USA Food and Drug Administration (FDA) (Basnet and Skalko-Basnet, 2011; Gupta et al., 2013). Curcumin doses from 4 to 8 g/day for 3 months in patients with preinvasive malignant or high-risk premalignant conditions have failed to produce any adverse effects (Cheng et al., 2001).

Quercetin, one of the most abundant dietary flavonoids, occurs naturally in a wide range of fruits and vegetables, such as red grapes, citrus fruits, tomatoes, broccoli, and green leafy vegetables. Quercetin supplements are widely used for improving immunity and maintaining general well-being. Extensive *in vivo* studies have revealed the anti-inflammatory, antiviral, immunoprotective, and antioxidant effects of quercetin (Nair et al., 2002; Robaszkiewicz et al., 2007; Uchide and Toyoda, 2011; Colunga Biancatelli et al., 2020; Salehi et al., 2020). Studies have also shown quercetin as cardioprotective, anticancer, antitumor, antiulcer, antiallergy, antidiabetic, gastroprotective, antihypertensive, and anti-infective/antimicrobial agent (Lakhanpal and Rai, 2007; Salehi et al., 2020; Yang et al., 2020). Quercetin is also a broad-spectrum antiviral and has shown inhibitory activity against many viruses including HIV, herpes simplex virus (type 1 and 2), poliovirus (type 1), parainfluenza (type 3), hepatitis C virus, human respiratory syncytial virus, Sindbis virus, vaccinia virus, SARS-CoV-1, and SARS-CoV-2 (Andersen et al., 1991; Kim et al., 2001; Semple et al., 2001; Yi et al., 2004; Gonzalez et al., 2009; Abian et al., 2020; Lopes et al., 2020; Rizzuti et al., 2021; Bahun et al., 2022). Quercetin has a well-established safety profile and doses up to 1 g/day for 3 months have not resulted in any significant adverse effects (Harwood et al., 2007; Andres et al., 2018). The USA FDA has approved quercetin as a safe agent (GRAS status) in dietary products for human use.

Vitamin D3 deficiency (VDD) is considered as a possible risk factor for susceptibility to COVID-19 infection and/or progression to severe illness. A number of published studies have reported significantly low serum vitamin D3 i.e. (1α,25(OH)_2_D3 levels in severely-ill COVID-19 patients, implying a possible association between VDD and the development of severe COVID-19 illness (Ilie et al., 2020; Meltzer et al., 2020; Mercola et al., 2020; Merzon et al., 2020). The precise role of vitamin D3 in COVID-19 remains uncertain. Vitamin D3 has well-documented immunomodulatory and anti-inflammatory effects, particularly in viral infections (Prietl et al., 2013; Tamer and Mesçi, 2013; Grant et al., 2020; Charoenngam et al., 2021). Due to the extreme toll of COVID-19 on the immune system, there has been a significant interest in the potential of vitamin D3 supplementation to ameliorate or prevent detrimental immune responses in the early stage of COVID-19 infection (Charoenngam et al., 2021).

The demonstrated anti-inflammatory (immunomodulatory), antiviral, and antioxidant activities of CQC, prompted us to investigate a combination of these agents as an adjuvant therapy for early-stage COVID-19 infection. In the present study, we conducted a pilot open-label, randomized controlled clinical trial to assess the possible treatment benefits of an oral formulation containing CQC supplements as an add-on to routine care in mild to moderately symptomatic COVID-19 outpatients. It is speculated that the co-supplementation of CQC in a single combined formulation could produce a more effective treatment, resulting from the synergistic anti-inflammatory (immunomodulatory), antiviral, and antioxidant effects of CQC. The synergistic mechanisms can effectively target the early stage of SARS-CoV-2 infection. This can be achieved by interfering with SARS-CoV-2 viral replication and simultaneously modulating the host’s hyperinflammatory response.

### Patients and Methods

This was a pilot, single-center, open-label, randomized controlled clinical trial conducted at the Department of Medicine, King Edward Medical University (KEMU), Lahore, Pakistan. The study was conducted according to the guidelines of the Declaration of Helsinki and informed written consent in the local language was obtained from each participant. The study was approved by the Institutional Review Board of KEMU (Approval No. 785/RC/KEMU) and registered at clinicaltrials.gov (registration No. NCT05130671).

Patients were enrolled at Mayo hospital, KEMU, Lahore COVID-19 outpatients’ clinic between 2 September 2021, and 28 November 2021. The inclusion criteria were: male or female aged ≥18 years; confirmed SARS-CoV-2 infection by reverse transcriptase polymerase chain reaction (RT-PCR)-based positive nasopharyngeal/oropharyngeal swab, typical COVID-19-associated acute symptoms including fever, a new and continuous dry cough, pharyngitis, myalgia, dyspnea (but not needing supplementary oxygen and SpO_2_ ≥ 93%), and asthenia, the need to see a physician as an outpatient for the acute symptoms, but not yet so severely-ill to require hospital admission. The exclusion criteria were: proven hypersensitivity or allergic reaction to quercetin or curcumin; on anticoagulant/antiplatelet therapy such as coumarin, heparin, aspirin, clopidogrel, dalteparin, enoxaparin, ticlopidine, and warfarin; diagnosed chronic kidney disease with estimated creatinine clearance <50 ml/min, or the need for dialysis; severe hypotension defined as needing hemodynamic pressors to maintain blood pressure; gallstone obstruction; hypothyroidism or moderate to severe thrombocytopenia (platelet count <100 × 10⁹/L).

### Randomization, Treatment, and Follow-Up

**FIGURE 1:**
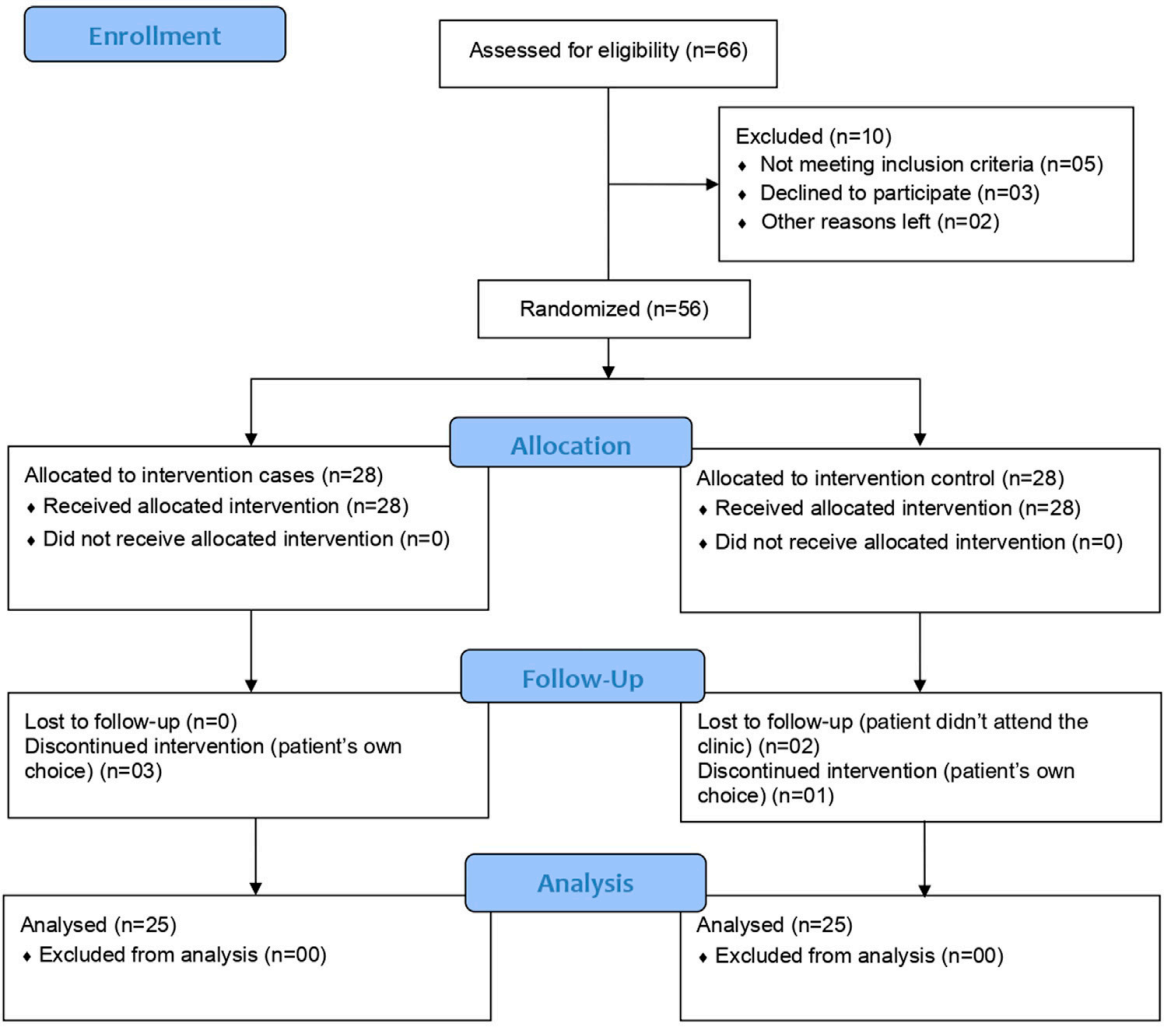
Study CONSORT flow diagram.

Patients presented with typical COVID-19-like symptoms at the Mayo Hospital, Lahore, COVID-19 outpatients’ clinic were first confirmed for SARS-CoV-2 infection by an RT-PCR analysis (Sansure Novel Coronavirus (2019-nCoV) Nucleic Acid Diagnostic Kit and PCR-Fluorescence Probing, Changsha, China). A total of 56 patients meeting all the inclusion criteria and none of the exclusion ones, consented and were randomized into the study in a 1:1 ratio to give 28 patients each in the CQC arm and control arm. Randomization was carried out using computer-generated random number tables with odd numbers allocated to the control arm and even numbers to the CQC arm patients. Of these 56, two patients from the control arm were lost to follow-up, three patients from the CQC arm, and another from the control arm discontinued the intervention on their own choice to leave a final study population of 50 patients, 25 in each arm (see below the Consolidated Standards of Reporting Trials (CONSORT) flow diagram in Figure 1). At enrolment (day one), participant’s acute COVID-19 symptoms and biochemistry including C-reactive protein (CRP) (Beckman Coulter^®^ CRP Latex, USA), D-dimer (Diazyme D-Dimer Assay, USA), lactate dehydrogenase (LDH) (Beckman Coulter^®^ LD, USA), ferritin (Access 2 Beckman Coulter^®^, USA), and complete blood count (CBC) (XN 1000, Sysmex, United Kingdom) were recorded at the outpatient’s clinic and treatment was prescribed by the physician as per the randomization. All patients received the COVID-19 standard of care (SOC) as per the hospital guidelines which include treatment with paracetamol with or without oral azithromycin (500 mg). In addition to the SOC medications, as an add-on, CQC arm patients were prescribed the adjuvant therapy as a single oral supplement formulation containing 42 mg of specific curcumin extract (Curt 001, 90% from *Curcuma longa* rhizome, Mumbai, India), 65 mg specific quercetin extract (Quer 001, ≥95% from *Sophora Japonica L.* whole flower bud, Changsha, China), and 2.25 µg (90 IU) cholecalciferol as two oral soft capsules b.i.d. (Nasafytol^®^, was kindly provided by TILMAN, Belgium) to be taken at home for 14 days. In total, the CQC adjuvants dosage consisted of daily supplementation of 168 mg of curcumin, 260 mg quercetin, and 9 µg (360 IU) of cholecalciferol.

Each patient had an in-person follow-up appointment with the outpatient’s physician on day seven of the treatment to check for the persistence of SARS-CoV-2 infection (using RT-PCR test of the nasopharyngeal/oropharyngeal swab), COVID-19-associated acute symptoms, and evaluation of the laboratory parameters. In case of patient not testing negative for SARS-CoV-2 on day seven, a second follow-up RT-PCR test was arranged for day 14, and if necessary, a third one on day 21.

### Primary and Secondary Outcomes

Patient testing negative for SARS-CoV-2 in the RT-PCR analysis was the primary outcome and improvement in the COVID-19-associated acute symptoms, and laboratory biochemistry were the secondary outcomes of this pilot study. Other outcomes included safety, tolerability, rate of hospitalization, length of hospitalization, duration of O_2_ support, rate of intensive care unit transfer, and mortality.

### Statistical Analysis

Continuous variables were compared between groups using the Welch two-sample *t*-test (normally distributed variables) and the Wilcoxon rank sum test (skewed variables). Binary and categorical variables were described as counts (and percentages) and compared between groups using the Chi-squared test or Fischer’s exact test when counts were sparse. The biomarkers showed skewed distributions. The effect of treatment on change from baseline in these biomarkers was first explored in simple scatter plots of day seven levels for individual patients against their baseline values overlaid with locally estimated scatterplot smoothing (LOESS) curves (Cleveland, 1979). The effect of randomized treatment within person change from baseline (or equivalently achieved levels adjusted for baseline values), was then formally tested by performing a non-parametric analysis of covariance (ANCOVA) using the ‘sm’ package in R (Young and Bowman, 1995; Bowman and Young, 1996; Bowman, 1997).

## Results

Table 1 below shows the patient’s baseline characteristics by treatment group. Overall, the median interquartile range (IQR) age was 44.0 (34.8, 52.0) years and included 25 (50.0%) males. The most common acute COVID-19 symptoms at enrollment were fever [40 (80.0%)], pharyngitis [26 (52.0%)], cough [27 (54.0%)], and myalgia [24 (48.0%)]. Less common symptoms were dyspnea and flu, each in seven (14.0%) patients. At entry, 33 (66.0%) patients had three or more COVID-19-associated symptoms, 13 (26.0%) had two symptoms, and four (16.0%) patients had a single symptom. A total of 21 (58.0%) patients had a history of pre-existing health conditions, and of these nine (36.0%) patients had a single, and 12 (24.0%) patients had two or more conditions. The most common comorbidities were diabetes mellitus (DM) and hypertension (HTN), found in 17 (34.0%), and 14 (28.0%) patients, respectively. Amongst the total 50 patients, 29 (52.0%) were COVID-19 vaccinated with at least two doses. The two treatment groups were comparable in terms of the proportion of men and the median (IQR) for age was only slightly lower in the CQC arm, i.e., 43.0 (32.0, 51.0) vs. 45.0 (37.0, 52.0) years of the control arm. The groups were similar in terms of numbers of COVID-19-associated symptoms; the number (%) of patients reporting three or more symptoms were 17 (68.0%) and 16 (64.0%) in the CQC and control arms, respectively. The control arm had slightly more COVID-19 vaccinated patients, i.e., 16 (64.0%) vs. 13 (52.0%) in the CQC arm, while the CQC arm had slightly fewer patients with DM and/or HTN, i.e., eight (32.0%) vs. 12 (48.0%) patients in the control arm. In both treatment groups, a similar number of patients had received antibiotic azithromycin as part of the SOC, i.e., 17 (34.0%) vs. 16 (32.0%) in the CQC and control arms, respectively. The randomized groups were considered reasonably balanced in terms of baseline characteristics.

**TABLE 1:** Demographics and baseline clinical characteristics of patients in the two treatment groups.

Characteristics	CQC Group (*n* = 25)	Control Group (*n* = 25)
Gender, male: n (%)	13 (52.0%)	12 (48.0%)
Age, years: mean (SD)	41.4 (12.9)	46.4 (12.4)
Age, years: median (IQR)	43.0 (32.0, 51.0)	45.0 (37.0, 52.0)
Age, years: (min, max)	(20.0, 66.0)	(23.0,76.0)
Age category (years)		
18–30	5	2
31–55	18	18
>55	2	5
Comorbidity	8 (32.0%)	13 (52.0%)
DM and/or HTN	8 (32.0%)	12 (48.0%)
Other	-	1 (4.0%)
Acute COVID-19 symptoms		
Fever	18 (72.0%)	22 (88.0%)
Pharyngitis	14 (56.0%)	12 (48.0%)
Cough	14 (56.0%)	13 (52.0%)
Myalgia	12 (48.0%)	12 (48.0%)
Flu	5 (20.0%)	2 (8.0%)
Dyspnea	2 (8.0%)	5 (20.0%)
≥3 COVID-19 symptoms	17 (68.0%)	16 (64.0%)
COVID-19 vaccination	13 (52.0%)	16 (64.0%)
Azithromycin in SOC	17 (34.0%)	16 (32.0%)

### Outcome of the Study

#### Primary Outcome

**FIGURE 2:**
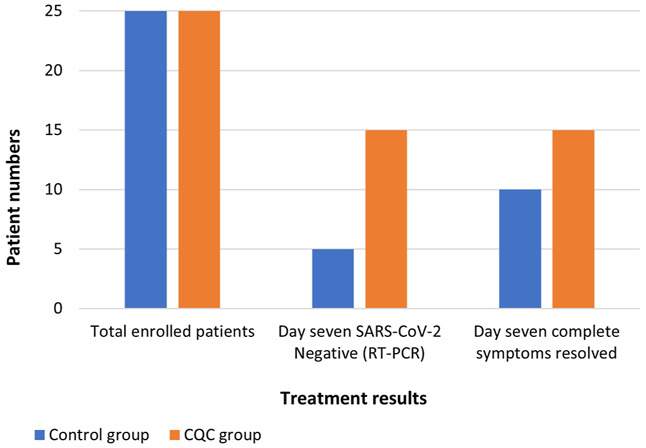
Comparison of RT-PCR SARS-CoV-2 test results and COVID-19 complete symptoms resolution observed at day seven in the two treatment groups.

Patients in the CQC arm cleared the SARS-CoV-2 infection faster with 15 (60.0%) of the CQC patients testing negative in the RT-PCR analysis by day seven compared to only five (20.0%) of the control arm patients (Figure 2); this treatment difference was statistically significant, *p* = 0.009. This difference in early viral clearance was not explained by differences in comorbidity; by repeating this comparison in those free of comorbidity at baseline, 11 (64.7%) of such CQC patients were clear of the virus by day seven compared to only one (8.3%) of the control arm patients, *p* = 0.003. Of the 10 CQC and 20 control arm patients still RT-PCR positive on day seven, all the CQC patients and all but one of the control arm patients tested negative by day 14, with this last remaining control arm patient eventually tested negative on day 21.

### Secondary Outcome

Upon assessment of the COVID-19-associated acute symptoms at follow-up day seven, symptoms had completely resolved in 15 (60.0%) of the CQC patients and the remaining 10 CQC patients were left with only one symptom (Figure 2). In comparison, symptoms had resolved in only 10 (40.0%) of the control arm patients, while 12 (48.0%) patients were left with one symptom, and three (12.0%) with two symptoms. This difference in the resolution of COVID-19 symptoms on day seven was consistent with a treatment benefit but not statistically significant, Fischer’s exact *p* = 0.154. Resolution of individual COVID-19 symptoms in the CQC and control arms, respectively were, 16/18 vs. 19/22 for fever (*p* > 0.9), 14/14 vs. 10/12 for pharyngitis (*p* = 0.203), 14/14 vs. 9/13 for cough (*p* = 0.041), 10/12 vs. 9/12 for myalgia (*p* > 0.9), 5/5 vs. 2/2 for flu, and 1/2 vs. 2/5 for dyspnea (*p* > 0.9).

**FIGURE 3:**
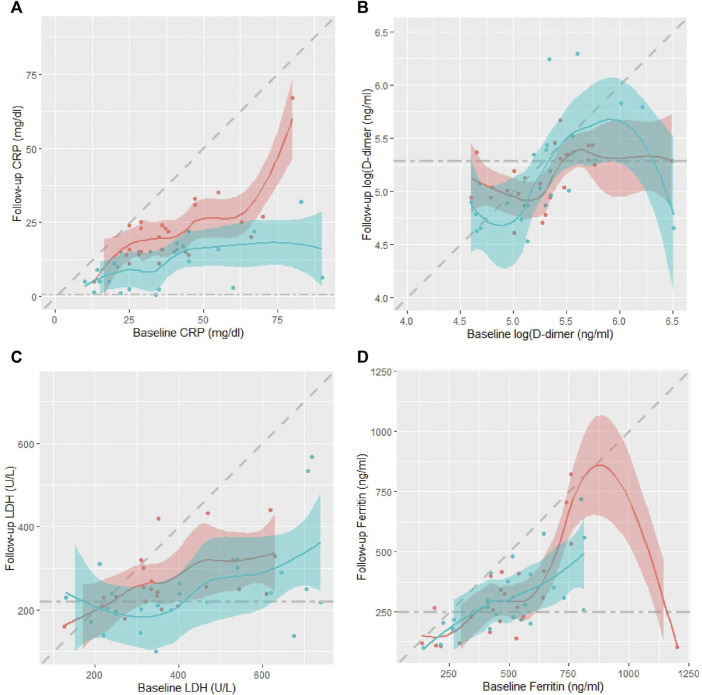
Scatter plots of individual patients’ day seven biomarker values against their corresponding baseline values in the two treatment arms, CQC (blue) and control (pink): **(A)** CRP, **(B)** D-dimer, **(C)** LDH, and **(D)** ferritin. Scatter plots overlaid with smoothed LOESS curves to help visualize any patterns. Diagonal dashed line denotes line of equality while horizontal dashed line represents the biomarker upper normal limit. The shading represents uncertainty around the smoothed lines between the points.

Table 2 shows patients’ serum inflammatory biomarkers levels at baseline and day seven follow-up. Despite similar levels at baseline, the median (IQR) CRP levels on day seven were significantly lower in the CQC arm compared to the control arm, only 11.0 (5.0, 16.0) mg/dl vs. 22.0 (15.0, 25.0) mg/dl, *p* = 0.0002 from a Wilcoxon rank sum test. Median D-dimer levels were almost identical at baseline in the two arms, and only slightly lower in the CQC arm on day seven, but not significantly so, *p* = 0.756, and in any case not by a clinically meaningful amount. LDH levels despite slightly higher values at baseline in the CQC arm, by day seven had fallen to levels below those for the control arm with some weak statistical evidence for a difference, *p* = 0.130. The serum ferritin levels despite slightly higher values at baseline in the CQC arm, by day seven had fallen to levels similar to those in the control arm. Figure 3 shows scatter plots of individual patients’ day seven biomarker values against their corresponding baseline values overlaid with smoothed LOESS curves through the points to help visualize any patterns; the shading represents uncertainty around the smoothed lines. The colors blue and pink represent patients in the CQC and control arms, respectively. Points below the diagonal line suggest that values have fallen between baseline and follow-up and that the patient’s biomarker profile has improved. In the CRP plot (Figure 3A), the values showed a clear separation of the curves for the two treatment arms. Also, the shaded line plot for CQC mostly sits below that for the control arm, suggesting better recovery with CQC adjuvant therapy. Other biomarkers values showed no such clear separation between the two treatment arms. D-dimer values showed a highly skewed distribution with a wide range and considerable within-group variability. Unlike for CRP, D-dimer levels showed no consistent fall by day seven in either arm (Figure 3B). In both treatment arms, LDH values generally fall between baseline and follow-up (Figure 3C). For ferritin, the values in the CQC arm were generally lower at follow-up, but outliers in the control arm made it difficult to see a clear pattern (Figure 3D). Table 2 also shows *p*-values from the results of the non-parametric ANCOVA test for a treatment effect on day seven values adjusted for baseline values, or equivalently on the change from baseline adjusted for baseline. The results were as follows: adjusted for baseline, achieved CRP levels on day seven were significantly lower in the CQC arm compared to the control arm, *p* = 0.006, but there was no evidence for a treatment effect on achieved levels of D-dimer, LDH, and ferritin, with test *p*-values of 0.169, 0.289, and 0.280, respectively.

**TABLE 2:** Serum inflammatory biomarker levels and hematology at baseline and follow-up day seven in the two treatment groups.

Biochemistry	Baseline (Day One)		Day Seven of Treatment		Test of Treatment Effect on Change
	CQC Arm	Control Arm	CQC Arm	Control Arm	*p*-value
CRP, median (IQR) (mg/dl)^a^	34.0 (21.0, 45.0)	36.0 (28.0, 47.0)	11.0 (5.0, 16.0)	22.0 (15.0, 25.0)	0.006
D-dimer, median (IQR) (ng/ml)^b^	202.0 (160.0, 259.0)	201.0 (150.0, 244.0)	153.0 (130.0, 219.0)	168.0 (140.0, 210.0)	0.169
LDH, median (IQR) (U/l)^c^	381.0 (250.0, 622.0)	335.0 (310.0, 465.0)	219.0 (196.0, 264.0)	243.0 (220.0, 301.0)	0.289
Ferritin, median (IQR) (ng/ml)^d^	495.0 (400.0, 590.0)	470.0 (413.0, 560.0)	269.0 (217.0, 378.0)	268.0 (165.0, 400.0)	0.280
**Hematology**					
Hemoglobin, median (IQR) (g/dl)^e^	11.9 (11.0, 13.0)	11.0 (11.0, 12.0)	11.0 (10.1, 12.5)	11.0 (10.8, 12.0)	0.622
Platelets, median (IQR) (×10^9^ cells/l)^f^	244.0 (230.0, 327.0)	301.0 (230.0, 400.0)	310.0 (255.0, 350.0)	310.0 (234.0, 350.0)	0.148
Lymphocytes, median (IQR) (×10^9^ cells/l)^g^	9.0 (7.1, 10.0)	11.0 (9.0, 11.6)	8.6 (7.0, 10.0)	9.5 (8.5, 12.0)	0.745
Leukocytes, median (IQR) (×10^9^ cells/l)^h^	36.0 (26.0, 43.0)	20.0 (14.0, 34.0)	24.0 (22.0, 25.0)	23.0 (17.0, 25.0)	0.680
Neutrophils, median (IQR) (×10^9^ cells/l)^i^	56.0 (53.0, 65.0)	71.0 (62.0, 76.0)	71.0 (70.0, 72.0)	71.0 (68.0, 74.0)	0.219

Normal reference levels:

a CRP: <0.50 mg/dl.

b D-dimer: up to 198 ng/ml.

c LDH: 120–220 U/L.

d Ferritin: 20–250 ng/ml.

e Hemoglobin: 13.5–17.5 g/dl (male); 12.15.5 g/dl (female).

f Platelets: 150–450 (× 10^9^ cells/l).

g Lymphocytes: 4.0–11.0 (× 10^9^ cells/l).

h Leukocytes: 1.5–4.5 (× 10^9^ cells/l).

i Neutrophils: 2–7.5 (× 10^9^ cells/l).

Furthermore, Table 2 also compares median blood cell counts in the two arms and tests for a treatment effect on the change from baseline to follow-up day seven. Hemoglobin count, similar in both arms at baseline, showed no noticeable change in either arm, *p* = 0.622. Platelet count at baseline was lower in the CQC arm compared to the control arm, but not significantly so, *p* = 0.734, rose during follow-up so that by day seven was identical to that for the control arm. However, there was only very weak statistical evidence for a treatment effect, *p* = 0.148. Lymphocyte count at baseline was only slightly lower in the CQC arm, but not by a clinically meaningful amount; day seven counts were very similar in the two arms with no noticeable change, *p* = 0.745. Leukocyte count at baseline was significantly higher in the CQC arm (*p* = 0.001) but fell so that by day seven count was similar to that in the control arm. Leucocytes count changed little in the control arm and there was no evidence for a treatment effect on change from baseline count (logged values), *p* = 0.680. Neutrophil count at baseline was lower in the CQC arm compared to the control arm (*p* = 0.0006) but rose so that by day seven counts were similar in the two arms. The median neutrophil count (logged values) in the control arm showed no noticeable change, *p* = 0.219.

### Other Outcome

In both treatment groups, all the patients recovered from COVID-19 infection, and none progressed to severe illness at least during the 14 days study follow-up period. The adjuvant therapy of co-supplementation of CQC was well-tolerated by all 25 patients and no treatment-emergent effects, complications, side effects, or serious adverse events were reported.

## Discussion

This pilot randomized controlled clinical trial assessed the possible treatment benefits of CQC in a single oral formulation, as a complementary therapy to the routine care for early-stage mild to moderate symptoms of COVID-19 in outpatients. In this study, patients who received the tripartite co-supplementation of CQC as an adjuvant therapy, most (60%) of them cleared their SARS-CoV-2 viral infection faster (on day seven of the treatment), as compared to the control arm patients (only 20%). Similarly, more patients (60%) in the CQC arm showed their complete COVID-19 acute symptoms resolution by day seven of the treatment, as compared to the control arm (40%). It is speculated that the speedy clearance of the SARS-CoV-2 in the CQC treated patients may be due to the inhibition of 3C-like protease (3CLpro) also called main protease (Mpro) of the SARS-CoV-2 by curcumin and quercetin as reported by the *in vitro* studies (Chen et al., 2006; Abian et al., 2020; Bormann et al., 2021; Marin-Palma et al., 2021; Rizzuti et al., 2021; Bahun et al., 2022). 3CLpro is an essential enzyme in the SARS-CoV-2 replication cycle, processing the large viral polyproteins and rendering the individual proteins functional. 3CLpro is highly conserved amongst coronaviruses and is currently the main target for antiviral therapies in COVID-19. The expedited viral clearance of CQC patients could also be due to other antiviral mechanisms associated with curcumin and quercetin. Curcumin can exert antiviral effects directly on the viral particle or at different stages of the replicative cycle by interacting with viral proteins or by interfering with cellular processes or pathways crucial for viral replication (Mazumder et al., 1995; Richart et al., 2018; Jena et al., 2021). Quercetin has been shown to block cell entry of influenza virus, rhinovirus, and SARS-CoV-1 (Yi et al., 2004; Wu et al., 2015), and interferes with the replication of herpesviruses and adenoviruses (Chiang et al., 2003). Quercetin also targets viral polymerases and may disrupt replication *via* the inhibition of reverse transcriptase and integrase enzymes (Ono and Nakane, 1990). Quercetin has also been shown to alter the expression of 98 of 332 (30%) human genes encoding protein targets of SARS-CoV-2, thus potentially interfering with the functions of 23 of 27 (85%) of the SARS-CoV-2 viral proteins in human cells (Glinsky, 2020). The clinical course of SARS-CoV-2 infection is quite unpredictable and therefore suitable early antiviral therapy is believed to be the key in preventing the progression to severe illness. We can, therefore, infer that the adjuvant therapy of CQC has the potential to target and interfere with the early stages of the SARS-CoV-2 infection (replication) and may possibly help in the speedy recovery of patients.

In addition to faster viral clearance, the co-supplementation of CQC adjuvant therapy also significantly reduced the patients’ hyperinflammatory response as shown by the significantly lowered serum CRP levels (by day seven of treatment) in the CQC arm as compared to the control arm (*p* = 0.006). We speculate that this significant improvement in the inflammation of patients in the CQC arm may be due to the multiple diverse anti-inflammatory mechanisms reported for curcumin, quercetin, and cholecalciferol. Curcumin has been shown to decrease the secretion of various critical pro-inflammatory cytokines including IL-1, IL-2, IL-6, IL-8, IL-10, IL-11, IL-12, IL-17, tumor necrosis factor alpha (TNF-α), and interferon-gamma (INF-γ), and chemokines such as monocyte chemoattractant protein 1, macrophage inflammatory protein 1α, nuclear factor kappa-light-chain enhancer of activated B cells (NFĸB), cyclooxygenase, plasminogen activator inhibitor-1, and caspase-3 (Babaei et al., 2020; Zahedipour et al., 2020). Curcumin also reportedly inhibits SARS-CoV-2-induced cytokine storm *via* deactivation of the mitogen-activated protein kinases/NFĸB signaling in the lung and liver epithelial cells (Sharma et al., 2022). Similarly, quercetin also exhibits diverse anti-inflammatory mechanisms, including the inhibition of lipid peroxidation, pro-inflammatory mediators such as lipoxygenase and phospholipase A2 (Colunga Biancatelli et al., 2020), and the nucleotide-binding oligomerization domain, leucine rich repeat and pyrin domain containing protein 3 (NLRP3) inflammasome-mediated IL-1β production (Tozser and Benko, 2016), and diverse proinflammatory cytokines such as IL-1β, IL-6, IFN-γ, and TNF-α (Nair et al., 2002; Chen et al., 2016; Leyva-Lopez et al., 2016). Quercetin in combination with dasatinib has been shown to selectively eliminate virus-induced senescent cells, mitigate COVID-19-reminiscent lung disease, and reduce inflammation in SARS-CoV-2-infected hamsters and mice (Lee et al., 2021). In a meta-analysis study of randomized controlled trials by Mohammadi-Sartang et al. (2017), quercetin diet significant reduced CRP levels, supporting its role as an anti-inflammatory agent. Furthermore, the immunomodulatory role of vitamin D3 is well-known (Bouillon et al., 2019). Vitamin D3 stimulates the expression of both innate and adaptive immune function receptors in the respiratory tract (Gombart et al., 2005; Chen et al., 2013; Brockman-Schneider et al., 2014; Martineau et al., 2017; Greiller et al., 2019). It also supports the innate immunity through the production of several antimicrobial peptides such as cathelicidins, defensivins, and IL-37 and acts on the adaptive immunity by modulating IL-6, IFN-γ, TNF-α, thus help in reducing lung tissue damage caused by inflammation. Serum CRP level is a crucial indicator of systemic inflammation and an important prognostic marker in patients with COVID-19. Elevated serum CRP levels are negatively correlated with SpO_2_ and associated with aggravation in asymptomatic and mildly symptomatic cases, and mortality in severely-ill COVID-19 patients, demonstrating lung injury linked disease course (Ali, 2020; Sadeghi-Haddad-Zavareh et al., 2021). Thus, the expedited significant decrease in the CRP levels at day seven indicates a faster clinical recovery (healing) of patients in the CQC arm, as compared to patients in the control arm, showing sustained inflammation. In study by Meng et al. (2020), in Wuhan, China, involving asymptomatic hospitalized COVID-19 pneumonia patients, out of 58 patients, 55 patients (94%) had developed ground glass opacity of lungs as shown by computed tomography scan, indicating a significantly high prevalence of lung involvement. Moreover, studies have shown close association between lung lesions and serum CRP levels in the early stage of COVID-19 infection (Wang, 2020). Therefore, the significantly reduced CRP levels of patients in the CQC arm, may act as an indirect marker of the improved lung pathology (if any) possibly due to the therapeutic effects of CQC adjuvant therapy. Any intervention targeted to reduce lesions/scarring of the lung in patients with COVID-19 infection can improve quality of life in the long term. Therefore, we can speculate that the CQC add-on therapy tested in this study may provide long-term benefits to lung health.

The potential treatment benefits of CQC add-on therapy for COVID-19, as observed in this pilot clinical trial are in line with the results reported by another pilot randomized controlled clinical trials and case series studies that investigated curcumin, quercetin, and cholecalciferol acting either alone or in combination with other dietary supplements as an adjuvant treatment for patients with COVID-19. Studies investigated curcumin as an adjuvant for COVID-19 have revealed multiple therapeutic benefits including early negativization of the SARS-CoV-2, early symptomatic recovery, increased lymphocyte count, less clinical deterioration, shorter hospitalization period, reduce risk and duration of supplementary oxygen, mechanical ventilation, and mortality (Ahmadi et al., 2021; Chabot and Huntwork, 2021; Majeed et al., 2021; Pawar et al., 2021; Saber-Moghaddam et al., 2021; Asadirad et al., 2022). Several clinical trials, in particular, have investigated the anti-inflammatory and immunomodulatory pharmacological effects of curcumin in patients with COVID-19 and revealed improvement in serum CRP levels, a significant reduction in the gene expression, and/or serum levels of IFN-γ, IL-1β, IL-6, IL-10, IL-17, IL-21, IL-23, IL-35, retinoic acid-related orphan receptor-C, forkhead box P3, transforming growth factor beta, granulocyte-macrophage colony-stimulating factor, and frequency of T helper 17 cells (Salehi et al., 2020; Ahmadi et al., 2021; Tahmasebi et al., 2021a; Tahmasebi et al., 2021b; Hassaniazad et al., 2021; Asadirad et al., 2022). Similarly, several randomized clinical trials involving patients with COVID-19 have also revealed multiple treatment benefits for quercetin adjuvant therapy, including faster negativization of the SARS-CoV-2, early amelioration of the acute symptoms, reduction in the serum levels of inflammatory markers such as CRP, LDH, ferritin, and alkaline phosphatase (Kamel et al., 2020; Di Pierro et al., 2021a; Di Pierro et al., 2021b; Onal et al., 2021; Shohan et al., 2022). Moreover, with vitamin D3 adjuvant supplementation, early negativization of the SARS-CoV-2 and improvement in the acute symptoms have also been reported in patients with early-stage of COVID-19 (Opko Press Release, 2021; Sabico et al., 2021; Sanchez-Zuno et al., 2021; Rastogi et al., 2022). Vitamin D3, however, has shown no treatment benefits in patients with severe COVID-19 illness.

Taken together, there is a reasonable body of scientific and clinical evidence to suggest the safety and treatment benefits of curcumin, quercetin, and vitamin D3 for patients with COVID-19. Using a combination of these agents as an adjuvant therapy, the results observed in this pilot clinical trial suggest an expedited clearance of the SARS-CoV-2 viral infection and simultaneous modulation of the host hyperinflammatory response in patients with early mild to moderate symptoms of COVID-19. The faster viral clearance alongside a significant reduction in the acute inflammatory response with CQC adjuvant therapy is possibly due to the synergistic antiviral, anti-inflammatory, and antioxidant mechanisms of the three active agents. One potential serious risk of the currently used anti-inflammatory/immunomodulatory COVID-19 interventions such as corticosteroids, tocilizumab, or bariticinib, particularly in vulnerable and immunocompromised patients, is the immunosuppressive side effect of these drugs, which can further increase the risk of developing superimposed secondary bacterial or fungal infection, and thus progression to severe illness in these patients. In contrast, curcumin, quercetin, and vitamin D3 as anti-inflammatory agents have well-established individual safety profiles and do not suffer from the intrinsic drawback of immunosuppressive effects. Thus, their use as an adjuvant in combination with routine care, represents a safe therapeutic option for early-stage COVID-19 infection, particularly in vulnerable patients. Moreover, the antimicrobial activities of CQC could also prevent the development of such superadded or secondary infections in patients with COVID-19.

Our study was not free of limitations. In the present circumstances, particularly in developing countries, there is a desperate need for safe, cheap, accessible, and effective treatments for early-stage COVID-19 infection. Only clinical trials can identify such agents. The major limitations of our study were the small sample size, and not being a double-blinded and placebo-controlled trial. Large sample size studies are warranted to evaluate the results of our study.

## Conclusion

In conclusion, we have carried out a pilot open-label, randomized controlled clinical trial to assess the possible treatment benefits of an oral combination of curcumin, quercetin, and vitamin D3 supplements, in a single-formulation (CQC), as an adjuvant therapy for patients with early-stage mild to moderate symptoms of COVID-19. Our results suggest that the co-supplementation of CQC is a safe intervention and possibly has a therapeutic role in the early stage of COVID-19 including faster clearing of the SARS-CoV-2 viral infection, and rapid improvement of the acute symptoms, and modulation of the early-stage hyperinflammatory response. The synergistic effect of the anti-inflammatory, immunomodulatory, antiviral, and antioxidant activities of CQC is believed to be the driving force for faster COVID-19 recovery of patients observed in this pilot clinical trial. Considering the well-established safety profile, tolerability, worldwide over-the-counter availability, and low cost, the combination of curcumin, quercetin, and vitamin D3 supplements could be used as an adjuvant therapy for the early-stage mild to moderate symptoms of COVID-19 infection.

## Data Availability

The data supporting the findings of this study can be made available from the corresponding author upon reasonable request.
